# MECoRank: cancer driver genes discovery simultaneously evaluating the impact of SNVs and differential expression on transcriptional networks

**DOI:** 10.1186/s12920-019-0582-8

**Published:** 2019-12-30

**Authors:** Ying Hui, Pi-Jing Wei, Junfeng Xia, Yu-Tian Wang, Chun-Hou Zheng

**Affiliations:** 1 Key Lab of Intelligent Computing and Signal Processing of Ministry of Education, College of Computer Science and Technology, Anhui University, Hefei, China; 2 Institute of Physical Science and Information Technology, Anhui University, Hefei, China; 3 School of Software Engineering, Qufu Normal University, Qufu, China

**Keywords:** Driver genes, Cancer, Transcriptional networks

## Abstract

**Background:**

Although there are huge volumes of genomic data, how to decipher them and identify driver events is still a challenge. The current methods based on network typically use the relationship between genomic events and consequent changes in gene expression to nominate putative driver genes. But there may exist some relationships within the transcriptional network.

**Methods:**

We developed MECoRank, a novel method that improves the recognition accuracy of driver genes. MECoRank is based on bipartite graph to propagates the scores via an iterative process. After iteration, we will obtain a ranked gene list for each patient sample. Then, we applied the Condorcet voting method to determine the most impactful drivers in a population.

**Results:**

We applied MECoRank to three cancer datasets to reveal candidate driver genes which have a greater impact on gene expression. Experimental results show that our method not only can identify more driver genes that have been validated than other methods, but also can recognize some impactful novel genes which have been proved to be more important in literature.

**Conclusions:**

We propose a novel approach named MECoRank to prioritize driver genes based on their impact on the expression in the molecular interaction network. This method not only assesses mutation’s effect on the transcriptional network, but also assesses the differential expression’s effect within the transcriptional network. And the results demonstrated that MECoRank has better performance than the other competing approaches in identifying driver genes.

## Background

Recent advances in deep sequencing have provided us with an unprecedented amount of cancer genomics data. With the rapid accumulation of huge volumes of genomic data, we have tremendous opportunities to better understand cancer initiation, progression and development [1]. However, it is still a challenge to decipher those data (e.g., single nucleotide variants (SNVs), small insertions or deletions (indels), large copy-number variations (CNVs) and structural aberrations, etc.) and use them to distinguish driver mutations which contribute to cancer development from passenger mutations that have accumulated in somatic cells but without functional consequences [2, 3]. In fact, in the early stages, single data, such as somatic aberrations data is the most commonly used data to identify driver genes. For example, frequency-based methods, such as MuSiC [3] and MutSigCV [4], are the common approach which relies on the frequency of aberration of a given gene or locus in a population of tumors [5]. In addition, machine learning approaches based on alterations knowledge are also used to identify driver genes. For instance, CHASM adopts random forest which use alterations trained from known cancer-causing somatic missense mutations to classify driver mutations [6, 7]. Recently, many mathematical and statistical methods which are based on data integration were proposed to search for driver genes, driver pathways or core modules [8]. Bayesian network-based methods such as CONEXIC integrated copy number change and gene expression data to identify potential driver genes which are located in some amplified or deleted regions in tumors [9].

With the developing of the research of cancer, we have recognized that the development and progression of cancer can be promoted by driver mutations or gene perturbing signaling, regulatory or metabolic pathways [1]. Thus, several methods that use network and pathway to understand drivers have been proposed, e.g., MEMo [10] and Dendrix [11]. MEMo uses the mutual exclusivity of gene mutations to detect mutated subnetworks critical to carcinogenesis [10]. As well as Dendrix was designed to identify subnetworks with potential driver activity which have high coverage and high mutual exclusivity [11]. Another method, MUFFINN measures the functional impact of the network neighbors of mutated genes, and scores the investigated genes by considering the influence of either the most frequently mutated neighbor or all direct neighbors [12]. Although the aforementioned approaches have achieved great achievements in distinguishing driver genes, improving the identification accuracy of driver genes is still a challenge.

In this work, we propose a method named MECoRank to prioritize driver genes of single patient sample based on their impact on the expression in the molecular interaction. Our method not only assess mutation’s effect on gene expression, but also assess the differential expression’s effect within a transcriptional network. We first construct a bipartite graph model to formulate associations between expression and somatic SNVs using protein-protein interaction (PPI) network. A bipartite graph is a graph whose vertices can be partitioned into two subsets. In our work, vertices on the left partition of the bipartite graph correspond to individual gene expression status and vertices on the right partition represent individual mutated genes. And then an iterative process was used to propagate the effect of somatic SNVs and differential expression. After iteration, we will obtain a ranked gene list for each patient sample. Finally, we applied the Condorcet voting method to determine the most impactful drivers in a population. To test the performance of our approach, we analyzed three datasets which are breast cancer dataset (BRCA), kidney renal clear cell carcinoma (KIRC) and lung squamous cell carcinoma (LUSC). From TCGA Data Portal (https://portal.gdc.cancer.gov/), we collected the data of somatic SNVs. And from UCSC [13], we obtained gene expression data. Experimental results show that our method not only can identify more driver genes that have been validated than other methods, but also can recognize some impactful novel genes. Although these genes are not presented in Cancer Gene Census (CGC), some evidences show that these candidate genes have functional roles in cancer or cancer-related biological processes.

## Methods

### Method overview

**Fig. 1:**
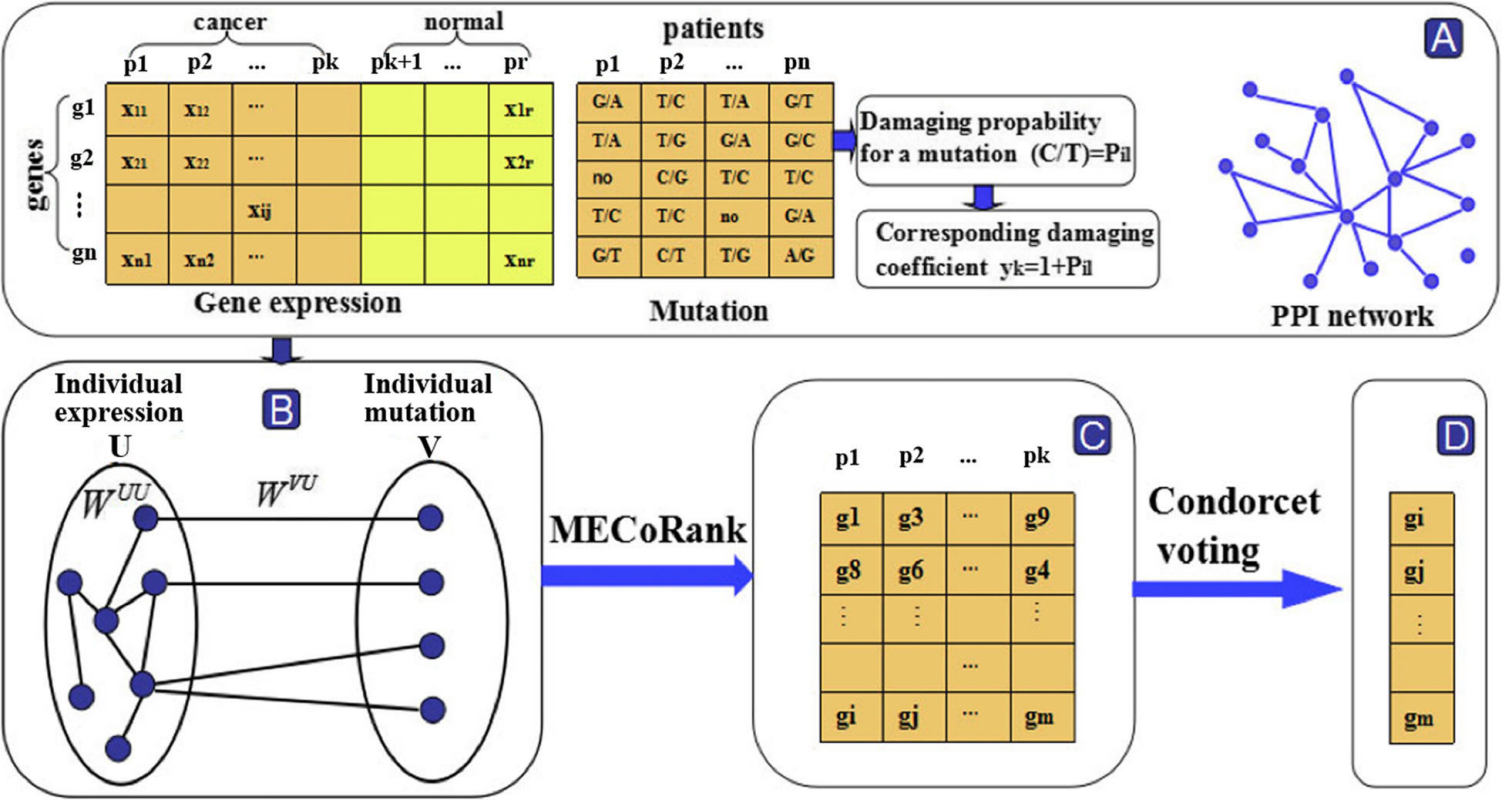
A schematic of MECoRank framework. **a** The data we used including gene expression of cancer and normal patients, somatic SNVs and PPI network. **b** The left partition of bipartite graph represents an individual patient’s expression set U where the transition matrices W^UU^ indicates the relations in U. The right of the bipartite graph represents the patient’s mutation set V where the transition matrices W^VU^ indicates the interactions between U and V. **c** We can obtain a score matrix in which each gene of every patient has a score. **d** We used the Condorcet voting to obtain the final rank of the genes

The proposed MECoRank method prioritizes driver genes based on the impact of their mutations and differential expression on the expression in the molecular interaction during a single patient. An overview of its workflow is presented in Fig. 1. In the following section, we first present the bipartite graph model and then give the iterative framework. Finally, we introduce Condorcet voting method for rank aggregation.

### A bipartite graph model

In this section, we detail the bipartite graph model used in our work. Consider a bipartite graph *G* = (*U* ∪ *V*, *E*), where *U* and *V* represent the individual expression and mutation respectively. Each edge in *E* connects a vertex in *U* and one in *V*. Let *U* = {*u*_1_, *u*_2_, …, *u*_*m*_} and *V* = {*v*_1_, *v*_2_, …, *v*_*n*_} be the two sets of *m* and *n* genes. We use *u*_*i*_ to denote the *i* − *th* vertex in *U*, and *v*_*j*_ to denote the *j* − *th* vertex in *V*, where 1 ≤ *i* ≤ *m* and 1 ≤ *j* ≤ *n*. For each patient, an edge between the nodes on the left and right partitions of the graph is drawn if *u*_*i*_ and *v*_*j*_ are known to interact according to PPI network. We constructed the bipartite graph for each patient by the same way. The edges between *U* and *V* are represented as the transition probability *W*^*VU*^ [14]. And the edges within *U* are represented as *W*^*UU*^. If there is an edge connecting *u*_*i*_ and *v*_*j*_ in PPI, ${w}_{ij}^{vu}=1$; otherwise, ${w}_{ij}^{vu}=0$. As such, we can describe all edges weights of the graph as a *m* × *n* matrix ${W}^{VU}=\left[{w}_{ij}^{vu}\right]$. Similarly, we can easily obtain the transition matrix *W*^*UU*^. For each vertex *v*_*j*_, we denote its degree (sum of connected edges’ weights) as *d*_*j*_, and use a diagonal matrix *D*_*v*_ to denote the degrees of all vertices in *V*; and similarly, for *d*_*i*_ and *D*_*u*_. Note that in this paper, we deal with undirected bipartite graphs.

### Iterative framework

**Fig. 2:**
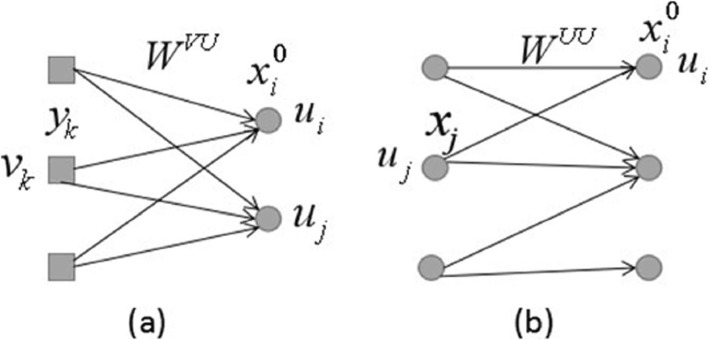
Score propagation on the bipartite graph: **a** score *y*_*k*_ represents the mutation corresponding damaging coefficient of *v*_*k*_. ${x}_i^0$ represents the value of tumor expression of *u*_*i*_. Score *y*_*k*_ is propagated to *u*_*i*_ and *u*_*j*_. **b** *x*_*j*_ represents the value of differential expression and score *x*_*j*_ is propagated to *u*_*i*_

To rank vertices based on the graph structure, seminal algorithms like PageRank [15] and HITS [16] have been proposed [17]. PageRank is an algorithm used by Google Search to rank websites in their search engine results [18]. This approach estimates the importance score of vertices as the stationary distribution of a random walk process – starting from a vertex, the surfer randomly jumps to a neighbor according to the edge weight [17]. HITS algorithm is similar to PageRank in some aspects. This method assumes each vertex has two roles: hub and authority [16]. If a vertex is linked by many vertices with hub score, this vertex has high authority and vice versa [17]. These two methods are focus on unipartite graphs. Our iterative framework references HITS, PageRank and their variants which are often used for web search. In this work, we constructed the bipartite graph for each patient where vertices on the left partition of the graph correspond to individual gene expression status and vertices on the right partition represent individual mutated genes. And we utilized the iterative process which is shown in Fig. 2 to propagate the scores on the bipartite graph.

The intuition behind the score propagation is the reinforcement to boost co-linked entities on the bipartite graph [14]. The scores of vertices should follow a smoothness convention, namely that: a vertex (on the one side of bipartite graph) should ranked high if it is connected higher-ranked vertices (on the other side of the bipartite graph) [17]. In our model, the impact of mutation damaging probability and differential expression are propagated to expression on the bipartite graph. The greater the impact is, the higher the gene ranks. In order to incorporate content information of somatic SNVs and differential expression in the bipartite graph, the generalized equations can be written as

1 $$ {x}_i=\left(1-{\lambda}_u\right){x}_i^0+{\lambda}_u\left(1-{\lambda}_v\right)\sum \limits_{k\in V}{w}_{ik}^{vu}{d}_k^{-\frac{1}{2}}{y}_k+{\lambda}_u{\lambda}_v\sum \limits_{j\in U}{w}_{ij}^{uu}{d}_j^{-\frac{1}{2}}{x}_j $$

where *λ*_*u*_ *ϵ* [0, 1] and *λ*_*v*_ *ϵ* [0, 1] are the personalized parameters and *λ*_*u*_ + *λ*_*v*_ = 1. In this paper, we set *λ*_*u*_ is 0.9 since the impact of mutation on gene expression network may be more significant than differential expression within the network. ${x}_i^0$ is the standardized expression value of the *i* − *th* gene and *y*_*k*_ denotes the mutation damaging coefficient. And *y*_*k*_ = 1 + *p*_*il*_ which occurring in the *i* − *th* sample of gene *l*, where *p*_*il*_ is the sum of the probabilities of damaging effects of all mutations in gene *l* of the *i* th sample and is calculated using the PolyPhen and SIFT scores from TCGA. The reason why adding 1 to *p*_*il*_ in *y*_*k*_ is that in case of there is no mutation in the gene, while this gene is very important in the network. *d*_*k*_ is the degree of gene *k* and *d*_*j*_ is the degree of gene *j*. In order to suppress some high-degree vertices whose mutation damage are not so significant, we applied Laplacian normalization on degree matrix. *x*_*j*_ denotes gene differential expression and is calculated using tumor samples genes’ expression subtracting the mean of normal samples gene’s expression. When vertices in the right of the bipartite graph have 0 edges with the left, the sum degree will be 0, arising to a divide-by-zero error. What’s more, this may mean that the zero-vertices have no effect on the left vertices. To address the problem, we remove the zero-vertices. Our method converges when there is no longer a significant update in the ranks. That is when the magnitude of the difference of the ranks between time *t* + 1 and the previous time point t falls below *ε*, which we set to 0.0001. Iteration also stops when no solution is presented after a maximum number of iterations, which we set as 100. The final scores of *x*_*i*_ can be obtained through an iterative updating process. From our empirical testing, we find that in most cases the scores can converge after about 9 iterations.

### Condorcet voting for rank aggregation

After iteration, we could obtain the rank of genes for each patient. To determine the most impactful drivers in a population, we applied the Condorcet voting method modified by DawnRank [6]. The Condorcet voting method is a voting scheme in which ‘voters’ vote for the best ‘candidate’ by submitting a rank-ordered list of candidate preferences [6]. And by comparing every possible pair of candidates A and B, the Condorcet method selects a winning candidate and then determines a ‘winner’ by comparing the number of voters that preferred A to B and vice versa. We applied the modified Condorcet method to the iteration results to determine aggregate rankings of genes in a patient population. A penalty heuristic δ, a number between 0 and 1 in modified Condorcet method was implemented to lower the ranking of a gene in a pairwise comparison that is not mutated.

2 $$ PairwiseWinner\left(A,B\right)=\left\{\begin{array}{l}A\kern0.5em if\delta (A)\times Rank(A)>\delta (B)\times Rank(B)\\ {}B\kern13em otherwise\end{array}\right. $$

where

3 $$ \delta (A)=\left\{\begin{array}{c}\delta \kern2.75em if\ A\kern0.5em is\ not\ mutated\\ {}1\kern4.75em if\ A\kern0.5em is\ mutated\end{array}\right. $$

The modified Condorcet method can be accessed from R package of condorcetRanking. We set the penalty heuristic δ to be 0.85 as DawnRank used. We selected the top-100 ranked candidates as the driver genes for a patient population.

## Results

We applied MECoRank to three TCGA datasets, BRCA, KIRC and LUSC. First, we compared our method with DriverNet [5] and MUFFINN [12] to show the effectiveness of our method. For MUFFINN, we use two different versions MUFFINN-DNMax and MUFFINN-DNSum in the comparison analysis. Then, we performed Gene Ontology (GO) term and KEGG pathway enrichment analysis for the higher ranked genes by using the OmicShare tools, a free online platform for data analysis (www.omicshare.com/tools). In addition, we also summarized the distribution of the top 100 candidate-driver genes in druggable genes databases to analyze whether they are clinically relevant genes or not.

### Datasets

We applied MECoRank to 973 BRCA samples, 334 KIRC samples and 486 LUSC samples. The datasets we used in this paper consist of gene expression data and coding region mutation data for three cancer types. For mutation data, MECoRank just evaluate the impact of SNP mutation damaging. We downloaded the somatic SNVs data of three datasets from TCGA data portal in the version gdc-1.0.0. According to the somatic SNVs data, we got the mutation matrix and the SNP mutation damaging matrix. The SNP mutation damaging matrix was calculated using PolyPhen and SIFT score. PolyPhen and SIFT score are predictiors of the harmful effect of a mutation occurring in DNA sequences [19]. For PolyPhen score, we used the key word ‘damaging’ to filter eligible genes, as for the SIFT score, ‘deleterious’ was used. If there are more than one mutations in different locus in a gene, we summed those scores as this gene’s final mutation damaging. The PPI network we used in MECoRank was Human Protein Reference Database (HPRD) (http://www.hprd.org).

To help evaluating the quality of our results, we obtained a list of 616 cancer genes (see Additional file 1) from the well-studied cancer gene database, CGC and the version is (09/26/2016) [20].

### The comparison with DriverNet and MUFFINN

**Fig. 3:**
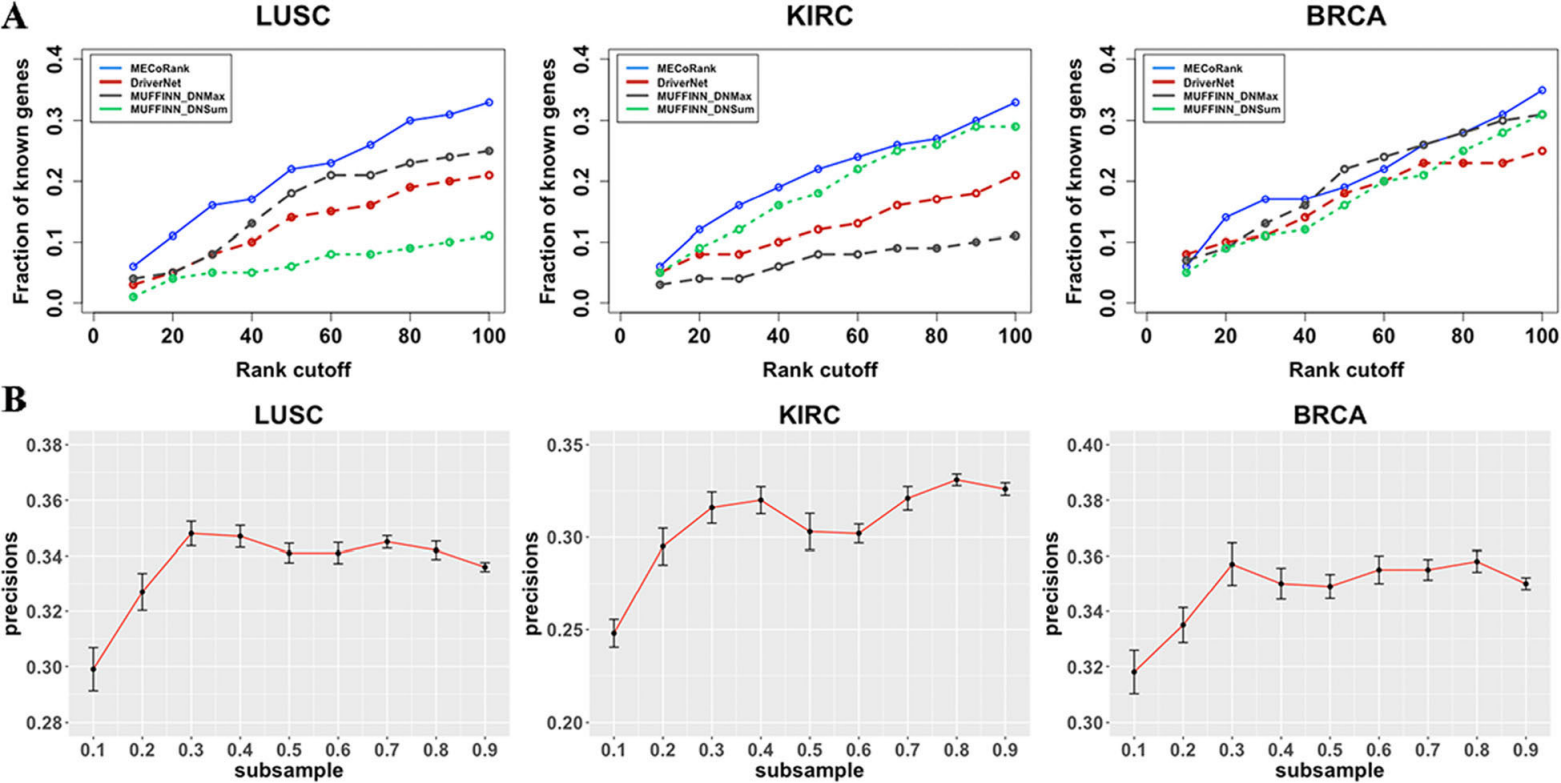
Performance comparision of driver gene predictions according to the cancer census genes set. **a** The performance of three methods (MECoRank, DriverNet, MUFFINN-DNMax and MUFFINN-DNSum) on LUSC, KIRC and BRCA datasets. **b** Precisions of MECoRank when we evaluate prediction from the CGC as a function of the size of the dataset

We evaluated the performance of three methods by examining the proportion of candidate-driver genes found in CGC. For DriverNet, we directly ran the package to find driver genes. And we ran MUFFINN in the online website. To facilitate the comparison, we applied the Condorcet rank for MECoRank result based on individual samples to provide the consensus population-level driver scores. The genes ranked within the top *k* score results were considerd as candidate driver genes. The proportion of known driver genes in the top *k* ranked results were calculated to obtain a criterion of performance evaluation called rank cutoff curves [21]. The range of *k* was set to [10,100] in interval 10. The rank cutoff curves of three methods were shown in Fig. 3a. In general, our method outperforms DriverNet and MUFFINN in all three cancer datasets with respect to CGC. On the other hand, to test the robustness of our method, we used a sub-sampling approach to evaluate the precisions of top 100 candidate-driver genes on BRCA, LUSC and KIRC [22]. The sub-sampling approach is the selection of a subset from cancer samples randomly to represent the population. We selected 10 to 90% samples from cancer samples as subset to run sub-sampling test. From the Fig. 3b, it can be seen that although the precisions are sensitive in small sample sizes, the precisions of our method are generally stable in three datasets. To assess more details of our methods on the precision, we adopt a measurement to denote the performance for predicting the driver genes, i.e. *P* =mean (*p*_*k*_), where *p*_*k*_ denotes the fraction of the top *k* (*k* = 1, 2, …, 100) predicted driver genes within the cancer census genes list [22]. The results of average precision of our method and comparison methods are listed in Table 1. From this table, we can see that our method outperforms DriverNet and MUFFINN in all three cancer datasets in general.

**Table 1:** The performance of our method and the other three comparison methods of the average precision in BRCA, KIRC and UCSC

	BRCA	KIRC	LUSC
MECoRank	0.544526362	0.55994818	0.523352895
DriverNet	0.526221857	0.390001192	0.305236274
MUFFINN_DNMax	0.533024334	0.244094534	0.325148101
MUFFINN_DNSum	0.44190754	0.472425214	0.193487171

When we applied MECoRank to all the three cancer datasets, we finally got a ranking list for each dataset, and the top 100 ranked mutations in the population were selected as the candidate-driver genes. The list of the 100 genes in three datasets is shown in Additional file 2.

**Fig. 4:**
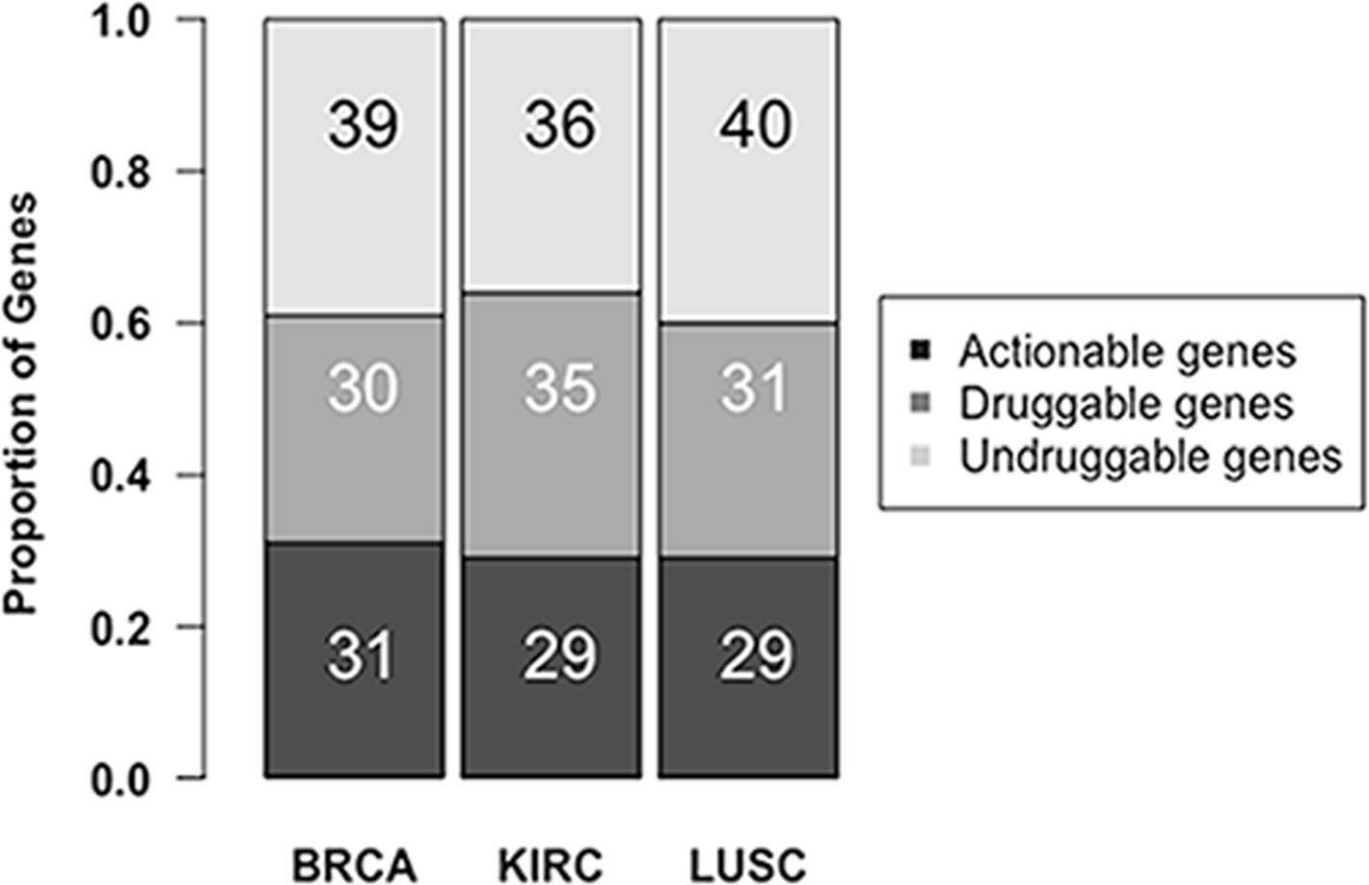
Distribution of three datasets’ top100 candidate-driver genes in druggable genes databases

For BRCA, there are 35 genes presented in CGC on the BRCA top 100 candidate-driver genes, which are more than DriverNet, MUFFINN-DNMax and MUFFINN-DNSum. Although MUFFINN-DNMax found 3 and 2 more genes in top rank 50 and 60 respectively, in general our method could find more driver genes. The top 10 ranking genes in BRCA are listed in Table 2. TP53, EP300, RB1, ESR1, CTNNB1 can be found in CGC. These five genes are known directly contribute to breast cancer progression according to IntOGen-mutational cancer drivers database [23]. COSMIC reported mutations in breast cancer most frequently in PIK3CA and TP53 while occasionally in CREBBP. However, a literature has pointed out that the gene CREBBP is also involved in the same function as Breast Cancer and Reported Genes by studying the function and pathway of the new gene [24]. Thus, we may conclude that CREBBP can also be said to be responsible for the disease. The rest four genes (YWHAG, ATXN1, UBQLN4, and SMAD9) also ranked high because of its high degree in transition probability *W*. Although they are not presented in CGC, some evidences show that these four candidate genes have functional roles in cancer or cancer-related biological processes. For example, miR-181b-3p promotes epithelial–mesenchymal transition in breast cancer cells through Snail stabilization by directly targeting YWHAG [25]. And ATXN1 is the target of miR-221 in regulating normal and malignant breast stem-like cells [26]. This is one of the few reports about ATXN1’s role in breast cancer. And for UBQLN4, there are few reports about it. But recently, a researcher found that a novel variant in UBQLN4 is associated with amyotrophic lateral sclerosis (ALS) and show that its expression compromises motor axon morphogenesis in mouse motor neurons and in zebrafish [27]. And SMAD9 is a new type of transcriptional regulator in bone morphgenetic protein signaling [28]. If SMAD9 have heterozygous mutations, it will cause heritable pulmonary arterial hypertension (HPAH), a serious lung vascular disease [29]. We also did Drug-Gene analysis on the top 100 genes of BRCA. We used the Drug-genes Interaction Database (DGIdb) online tool to analyze our data [30]. The results were shown in Fig. 4. Thirty-one BRCA candidate driver genes are found to be actionable targets. In addition, 30 other candidate driver genes are druggable.

**Table 2:** The top10 candidate driver genes in BRCA

Rank	Gene	Score	CGC gene
1	*YWHAG*	1	NO
2	*TP53*	0.999877321	YES
3	*CREBBP*	0.997637253	YES
4	*ATXN1*	0.997369452	NO
5	*EP300*	0.997363952	YES
6	*RB1*	0.996963919	YES
7	*ESR1*	0.996358809	YES
8	*UBQLN4*	0.995461351	NO
9	*SMAD9*	0.99541726	NO
10	*CTNNB1*	0.995314479	YES

For KIRC, there are 31 genes presented in CGC on the KIRC top 100 candidate-driver genes, which is more than DriverNet, MUFFINN-DNMax and MUFFINN-DNSum found. And from the rank cutoff curve, we can see that our method is better than the other methods in top 100 genes. The top 10 ranking genes are listed in Additional file 3. Among the top 10 genes, TP53, EP300, CTNNB1, CREBBP, SRC and AR can be found in CGC. We have pointed out that TP53, EP300, CTNNB1 and CREBBP are directly related to BRCA or other cancers. And SRC is human proto-oncogene, which was reported as a novel therapeutic target in renal cell carcinoma [31, 32]. The role of AR in KIRC progression is not clear, but it has been shown that in prostate and breast cancer cells AR binds to IGF1R promoter and thus increases IGF1R expression [33, 34]. The expression of IGF1R is inhibited by miRNA-223 [35] and miRNA-let-7i [36] that negatively associate with KIRC survival [37]. Although ATXN1, SMAD9, UBQLN4 and GRB2 are not included in CGC, we have already mentioned that ATXN1, SMAD9 and UBQLN4 are all relate to cancers or pathway in the last paragraph. As for GRB2, this gene participates in multiple cancer related pathway [31], such as chemokine signaling pathway, ErbB signaling pathway, MAPK signaling pathway and Jak-STAT signaling pathway. Among the top 100 genes in KIRC rank list, 29 genes are actionable targets and other 35 genes are at least druggable (Fig. 4).

For LUSC, there are 33 genes presented in CGC on the LUSC top 100 genes, but in DriverNet and two different MUFFINN version (MUFFINN-DNMax, MUFFINN-DNSum), they identified 21, 25 and 11 respectively. It shows that our method performs better. The top 10 ranking genes are given in Additional file 4. Among them, TP53, CREBBP, EP300, RB1, SMAD4 and ESR1 are presented in CGC. And of these six driver genes, TP53, RB1 and SMAD4 are directly related to LUSC according to IntOGen-mutational cancer drivers database [23]. ESR1 is known to play a very important role in cancer, and previous research found that ESR1 methylation is associated with concurrent methylation of a group of tumor suppressors [33]. And TGFBR1 involved in the transforming growth factor beta (TGF-β) signaling pathway had a significantly increased risk for cancer development [38]. The Drug-genes result (Fig. 4) shown that 29 genes are actionable targets and in addition 31 genes are druggable genes. From Fig. 4, we can see that almost 60% or more are druggable targets in top 100 genes in these datasets.

### Enrichment analysis

**Fig. 5:**
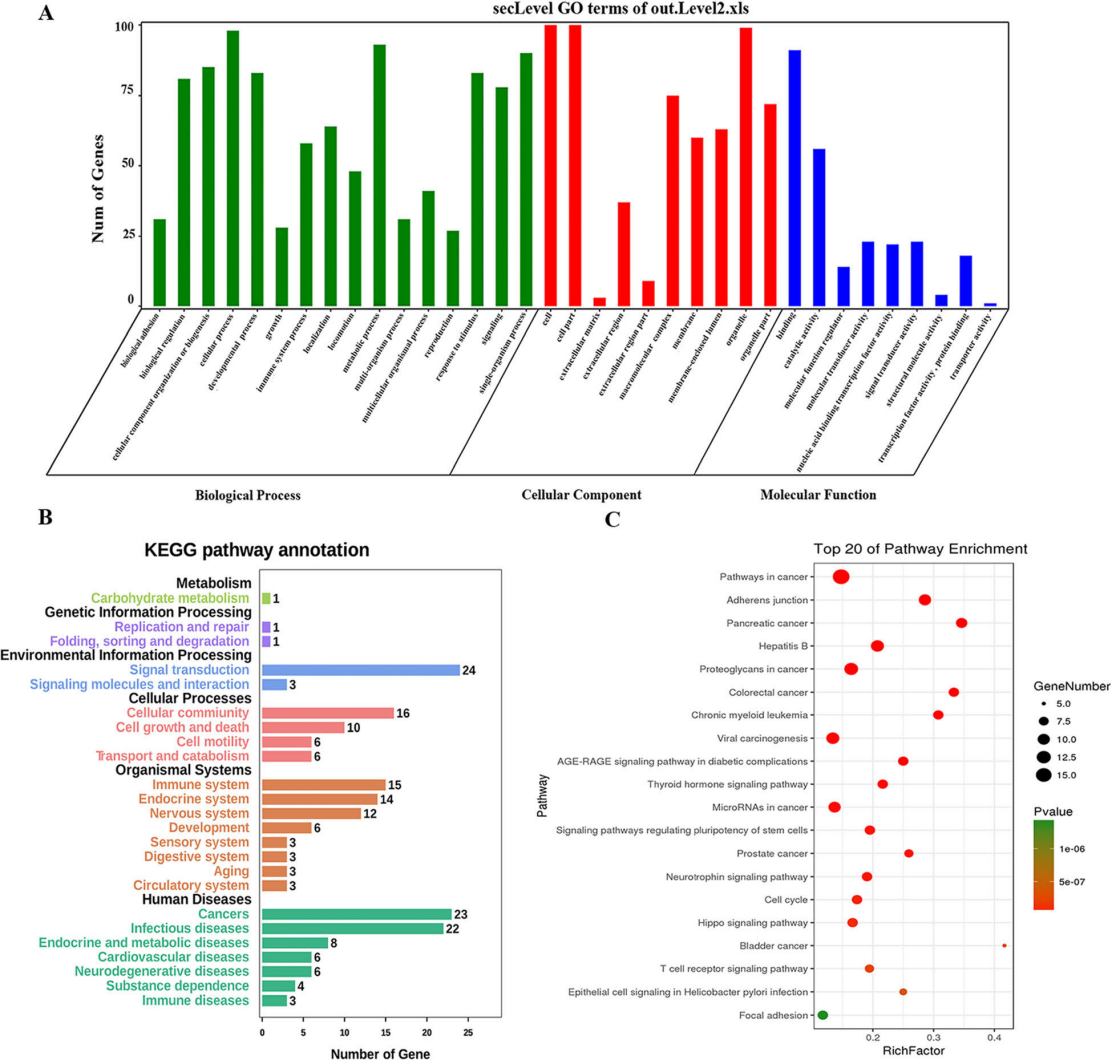
GO term and KEGG pathway enrichment analysis on BRCA rank list. **a** GO term enrichment analysis result of top100 candidate-driver genes in BRCA rank list. **b** KEGG pathway annotation result of top100 driver-candidate genes in BRCA. **c** Top 20 of pathway enrichment result of the top100 driver-candidate genes

To test whether the top 100 candidate-driver genes for the three investigated cancers are collaboratively working for particular biological functions or pathways, we performed Gene Ontology (GO) term and KEGG pathway enrichment analysis by using the OmicShare tools, a free online platform for data analysis (www.omicshare.com/tools). Here we only listed GO term enrichment analysis result on BRCA. The results of other two datasets were shown in Additional file 5. Go enrichment analysis revealed that the top100 candidate-driver genes of BRCA were significantly enriched in 35 GO terms which is shown in Fig. 5a. The most enriched GO terms were “cellular process” in the biological process, “cell” in the cellular components and “binding” in the molecular function. And the KEGG pathway results were shown in Fig. 5b-c. In Fig. 5b, the ordinate is the A level and the B level annotation of KEGG, the black typeface is the A level annotation name, and the color font is the B level. From this figure we can see that the largest number of genes enriched pathway is Signal transduction in Environmental Information Processing. And the most significantly enriched pathway in Human Diseases is Cancers. According to the pathway enrichment table, we selected the top 20 most significant pathways and displayed them in Fig. 5c. Among the top 20 pathways, the most significant pathway is Pathways in cancer. That means the top 100 genes in BRCA we identified were significantly related with cancer. Other pathways (e.g., Pancreatic cancer, Proteoglycans in cancer, Colorectal cancer and so on) were related to other cancer. What’s more, Hippo signaling pathway plays crucial roles in organ size control and cancer development. And it can interplay with mevalonate to regulate RHAMM transcription via YAP to modulate breast cancer cell motility [39].

## Discussion and conclusions

In this work, we proposed a method named MECoRank to prioritize driver genes of single patient sample based on their impact on the expression in the molecular interaction. The important contribution of our MECoRank is that we not only assess mutation’s effect on gene expression network, but also measure the differential expression’s effect within gene expression network. We applied MECoRank to three datasets (BRCA, KIRC and LUSC) which were obtained from TCGA and UCSC. Through evaluation of the benchmarking driver genes, MECoRank detected more known cancer driver genes than DriverNet and two different MUFFINN versions. That means MECorank yielded better performances than the other competing approaches.

However, there are also some limitations in our work. One is that the network we used is not complete, which will affect the construction of bipartite graph and eventually affect the result. So in the following work we will to construct a more completely network or use other methods to construct the bipartite graph. What’s more, we will try to integrate more information like CNVs and gene fusion by using a more completely network.

## Supplementary information

**Additional file 1:** A list of 616 cancer genes from Cancer Gene Census (CGC, 09/26/2016).

**Additional file 2:** A list of top100 candidate-driver genes of three datasets (BRCA, KIRC, LUSC).

**Additional file 3:** The table of top 10 ranking genes on KIRC datasets.

**Additional file 4:** The table of top 10 ranking genes on LUSC datasets.

**Additional file 5:** GO term enrichment analysis results on KIRC and LUSC.

## Data Availability

All data generated or analyzed during this study are included within article and its additional files.

## Abbreviations

ALS: Amyotrophic lateral sclerosisv
BRCA: Breast cancer dataset
CGC: Cancer Gene Census
CNVs: Copy-number variations
DGIdb: Drug-genes Interaction Database
GO: Gene Ontology
HPAH: Heritable pulmonary arterial hypertension
HPRD: Human Protein Reference Database
indels: Small insertions or deletions
KIRC: Kidney renal clear cell carcinoma
LUSC: Lung squamous cell carcinoma
PPI: Protein-protein interaction
SNVs: Single nucleotide variants
TGF-β: Transforming growth factor beta
