# Supplementary material for: MECoRank: cancer driver genes discovery simultaneously evaluating the impact of SNVs and differential expression on transcriptional networks
Source: BMC Med Genomics. 2019 Dec 30;12(Suppl 7):140. doi: 10.1186/s12920-019-0582-8 (PMC6936061; doi:10.1186/s12920-019-0582-8)
Supplement: Supplementary file 5 — Additional file 5: GO term enrichment analysis results on KIRC and LUSC. [file 12920_2019_582_MOESM5_ESM.pdf]

GO term enrichment analysis results on KIRC and LUSC

We did GO term and KEGG pathway enrichment analysis on KIRC and LUSC rank list. The results were shown in Figures 8 and 9. For the top 100 genes of KIRC, most gene-enriched GO terms were cellular process, cell, cell part, binding and so on. Pathway enrichment analysis revealed that well-known cancer pathways (e.g., cell cycle, focal adhesion) play critical roles in clear cell renal cell development [1]. The rest of other pathways (e.g., Proteoglycans in cancer, Bladder cancer, Pancreatic cancer and so on) were mostly related to other cancer and most genes were enriched in Pathways in cancer. For the top100 genes of LUSC, most gene-enriched GO terms were similar to KIRC and the most significantly enriched pathway were also Pathways in cancer. Available data indicated that Wnt signaling substantially impacts non-small cell lung cancer (NSCLC) tumorigenesis, prognosis, and resistance to therapy [2]. NSCLC includes lung squamous cell carcinoma [3]. And TGF-β signaling also plays an important role in the development of squamous cell carcinomas [4]. Other pathways also play important role in tumorigenesis.

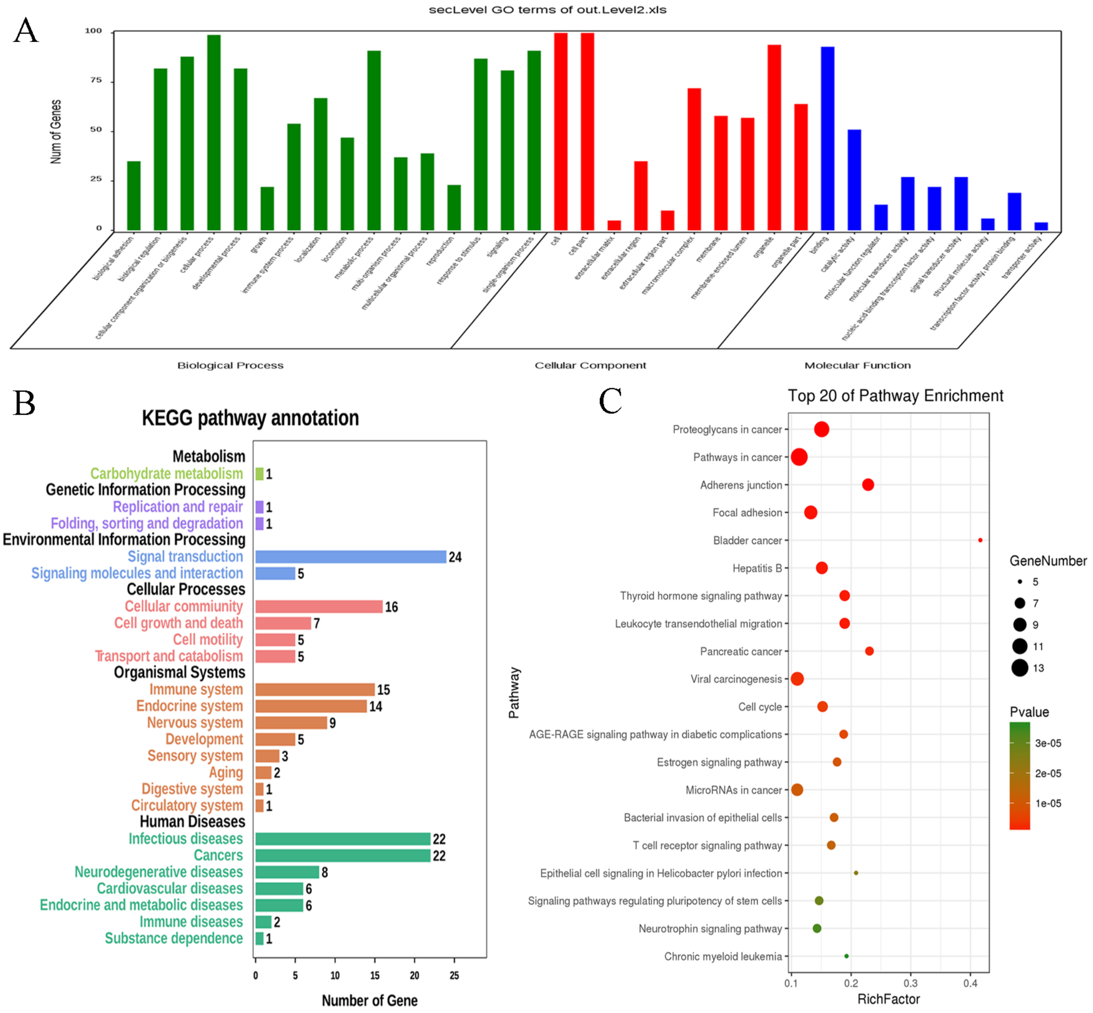

Fig. 1: GO term and KEGG pathway enrichment analysis on KIRC rank list.

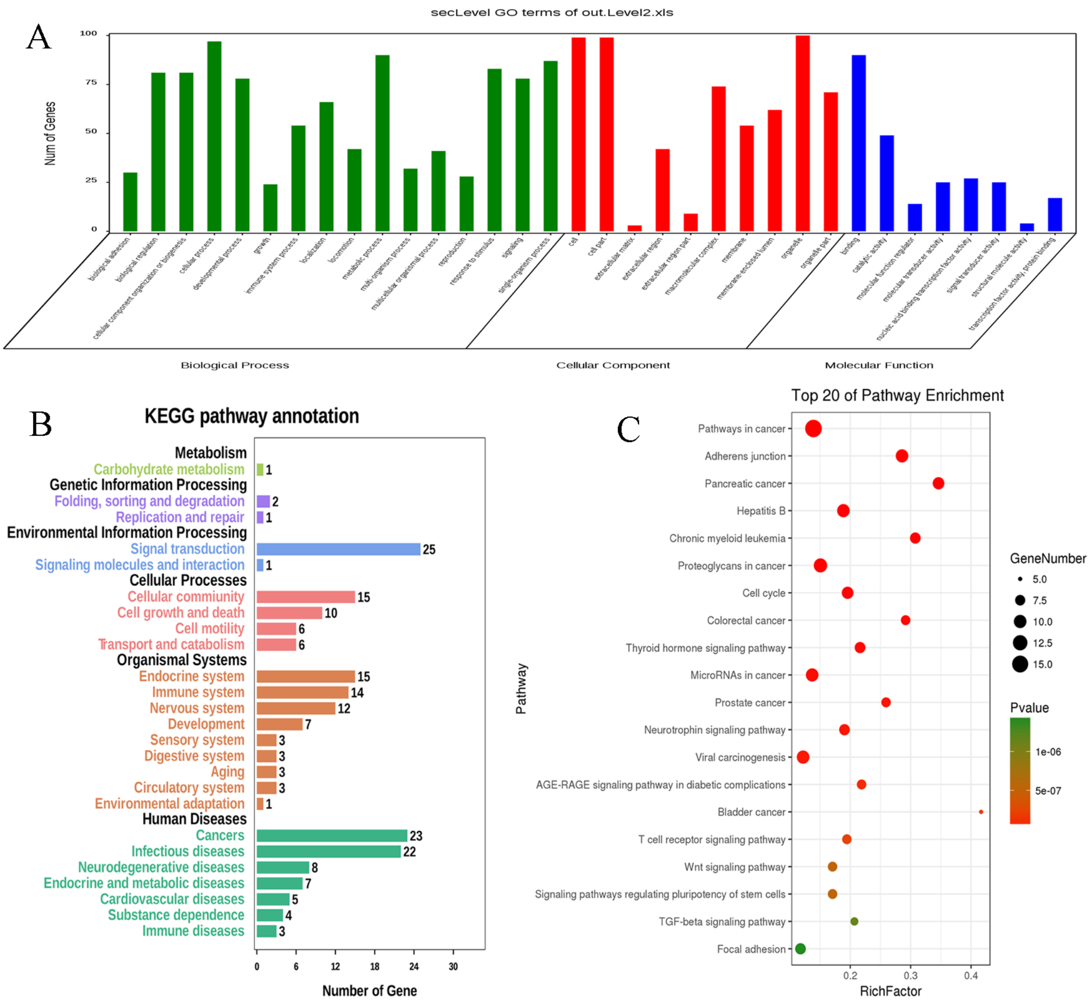

Fig. 2: GO term and KEGG pathway enrichment analysis on LUSC rank list.

1. Zhou, L., et al., *Integrated profiling of microRNAs and mRNAs: microRNAs located on Xq27. 3 associate with clear cell renal cell carcinoma.* PloS one, 2010. **5**(12): p. e15224.
2. Stewart, D.J., *Wnt signaling pathway in non–small cell lung cancer.* JNCI: Journal of the National Cancer Institute, 2014. **106**(1).
3. Uematsu, K., et al., *Activation of the Wnt pathway in non small cell lung cancer: evidence of dishevelled overexpression.* Oncogene, 2003. **22**(46): p. 7218.
4. Qiu, W., et al., *Disruption of transforming growth factor β-Smad signaling pathway in head and neck squamous cell carcinoma as evidenced by mutations of SMAD2 and SMAD4.* Cancer letters, 2007. **245**(1-2): p. 163-170.
